# An Antibody-Immobilized Silica Inverse Opal Nanostructure for Label-Free Optical Biosensors

**DOI:** 10.3390/s18010307

**Published:** 2018-01-20

**Authors:** Wang Sik Lee, Taejoon Kang, Shin-Hyun Kim, Jinyoung Jeong

**Affiliations:** 1 Hazards Monitoring Bionano Research Center, Korea Research Institute of Bioscience and Biotechnology (KRIBB), 125 Gwahak-ro, Yuseong-gu, Daejeon 34141, Korea; wang3026@kribb.re.kr (W.S.L.); kangtaejoon@kribb.re.kr (T.K.); 2 Department of Nanobiotechnology, KRIBB School of Biotechnology, University of Science and Technology, Daejeon 34113, Korea; 3 BioNano Health-Guard Research Center, Global Frontier Project, 125 Gwahak-ro, Yuseong, Daejeon 34141, Korea; 4 Department of Chemical and Biomolecular Engineering, Korea Advanced Institute of Science and Technology (KAIST), 291 Daehak-ro, Yuseong-gu, Daejeon 34141, Korea; kim.sh@kaist.ac.kr

**Keywords:** silica, inverse opal, optical biosensor, influenza virus

## Abstract

Three-dimensional SiO_2_-based inverse opal (SiO_2_-IO) nanostructures were prepared for use as biosensors. SiO_2_-IO was fabricated by vertical deposition and calcination processes. Antibodies were immobilized on the surface of SiO_2_-IO using 3-aminopropyl trimethoxysilane (APTMS), a succinimidyl-[(N-maleimidopropionamido)-tetraethyleneglycol] ester (NHS-PEG_4_-maleimide) cross-linker, and protein G. The highly accessible surface and porous structure of SiO_2_-IO were beneficial for capturing influenza viruses on the antibody-immobilized surfaces. Moreover, as the binding leads to the redshift of the reflectance peak, the influenza virus could be detected by simply monitoring the change in the reflectance spectrum without labeling. SiO_2_-IO showed high sensitivity in the range of 10^3^–10^5^ plaque forming unit (PFU) and high specificity to the influenza A (H1N1) virus. Due to its structural and optical properties, SiO_2_-IO is a promising material for the detection of the influenza virus. Our study provides a generalized sensing platform for biohazards as various sensing strategies can be employed through the surface functionalization of three-dimensional nanostructures.

## 1. Introduction

Influenza is an acute infectious disease caused by the influenza virus. The virus, which belongs to a genus of the *Orthomyxoviridae* family, is divided into three types: influenza A, B, and C [1]. The influenza A virus has recurrent epidemics and is recognized as a serious public health hazard [2]. A rapid and precise diagnosis is therefore important to prevent the spread of the disease. Existing virus detection techniques, such as enzyme-linked immunosorbent assay (ELISA) or polymerase chain reaction (PCR), have drawbacks, such as requiring time-consuming and specialized processes [3,4,5,6]. Therefore, a technique that is convenient, rapid, sensitive and selective is required for the detection of pandemic viruses. Recently, diverse integrated optical biosensors, based on interferometers, grating couplers, microring resonators, photonic crystals, or micro/nanophotonics transducers, have been studied for the detection of various bio-markers [7,8]. These techniques can be utilized to produce highly-sensitive and ultra-compact biosensors using light–matter interactions [9,10]. Additionally, because integrated optical biosensors have the advantage of direct, real-time, and label-free detection, they are appropriate for the detection of biohazards such as viruses and bacteria. Among integrated optical biosensors, photonic crystals (PCs) are able to make interesting biosensors due to interaction of structural properties and light.

PCs, as optical materials with a periodic nanostructure, have attracted much attention due to their unique optical property known as the photonic band gap (PBG) [11]. A PBG is a specific range of wavelengths in which light propagation is forbidden [12,13,14]. The wavelength and width of PBG are determined by the periodicity and refractive index of the nanostructures. Since PCs were intensively studied by John [15] and Yablonovitch [16] in 1987, various PCs have been developed by top-down and bottom-up approaches [17]. PCs have diverse applications, including in displays [18], lasers [19], and sensors [20]. In particular, PC-based plasmonic sensors have been reported to be effective for virus detection [21,22]. However, these sensors have limitations. For example, only metal surfaces can be used as substrates, and highly expensive processes, such as e-beam lithography, are required [23]. Among PC nanostructures, inverse opals (IOs) are three-dimensionally ordered porous structures with a regular arrangement of spherical cavities in a solid matrix, assembled through colloidal self-assembly. IO nanostructures are fabricated from an opal template which is composed of closely-packed spherical particles in a face-centered cubic (FCC) lattice [24,25]. The optical properties of IO nanostructures are determined by the lattice constant and refractive index of the cavities and matrix. Because these factors can be tuned by external stimuli, IO nanostructures can be used as sensing materials for the detection of target molecules [26]. Previous studies of IO-based sensors generally focused on the detection of small molecules or changes in environmental conditions. For example, Li et al. demonstrated a TiO_2_-based IO nanostructure as a label-free immunosensor in which the diffraction peak was shifted by physical adsorption on the porous surface. This system is limited by its non-specific binding and low sensitivity [27]. Jiang et al. suggested an enzyme-based IO structure for biocatalysis. This IO nanostructure showed better stability in alkaline pH and at high temperatures [28]. In addition, Couturier et al. reported a hydrogel-based IO sensor for the recognition of systems such as lectins by specific sugars and avidin by biotin. The molecules were detected by tuning the lattice spacing caused by structural changes such as swelling and shrinking [29]. Recently, an IO structure with a hydrogel backbone was used to successfully detect rotavirus by surface functionalization [30]. 

In this study, we report the detection of the influenza virus by SiO_2_-based IO (SiO_2_-IO) nanostructures. The fabrication of SiO_2_-IO nanostructures is a low-cost process providing a large surface area, high mechanical stability, and unique optical properties for utilization as biosensors. A silanized SiO_2_ surface was used for the immobilization of the influenza virus antibody by chemical and biological linkers. With the antibody-immobilized SiO_2_-IOs (Ab-SiO_2_-IOs), the influenza virus was selectively captured on the surface of the cavities, resulting in the redshift of the reflectance peak. Therefore, the influenza virus could be detected by simply monitoring the change in reflectance spectra without complicated labeling procedures. Our study demonstrated that SiO_2_-IO nanostructures are suitable for generalized biosensor applications as various sensing strategies can be potentially employed through the surface functionalization of the three-dimensional nanostructures.

## 2. Materials and Methods 

### 2.1. Materials and Reagents

To fabricate the IO nanostructures and perform surface functionalization, ethanol (EtOH) and methanol (MeOH) were purchased from EMD Millipore Co. (Billerica, MA, USA). Hydrochloric acid (HCl) was purchased from DUKSAN Co. (Ansan, Korea). Phosphate-buffered saline (PBS, pH 7.4, 1×) was purchased from Invitrogen Co. (New York, NY, USA), and hydrofluoric acid (HF, 49%) was purchased from J.T. Baker (Center Valley, CA, USA). Latex bead polystyrene, tetraethyl orthosilicate (TEOS), 3-aminopropyl trimethoxysilane (APTMS), and anhydrous toluene were purchased from Sigma-Aldrich (St. Louis, MO, USA). NHS-PEG_4_-Maleimide cross-linkers were purchased from Thermo Scientific (Waltham, MA, USA). Horseradish Peroxidase (HRP) conjugated anti-rabbit secondary antibody (Goat Anti-Rabbit IgG H&L (HRP)) was purchased from Abcam Co. (Cambridge, UK). Cystein-tagged protein G (Cys-ProG) was obtained from Bioprogen Co. (Daejeon, Korea). Bovine serum albumin was purchased from Santa Cruz Biotechnology (Dallas, USA). For detection of the virus, the hemagglutinin (αHA-1) antibody, the pandemic influenza A (H1N1) virus (A/CA/07/2009), the influenza A (H3N2) virus (A/canine/Korea/MV01/2012), and the influenza B virus (IFVB, B/Yamagata/Florida/04/06) were provided by BioNano Health Guard Research Center (H-GUARD). To functionalize the gold nanoparticles (AuNPs), gold (III) chloride trihydrate (HAuCl4) and sodium citrate were purchased from Sigma (St. Louis, MO, USA), and gold binding peptide-protein G (GBP-proG) was purchased from Bioprogen Co. (Daejeon, Korea).

### 2.2. Preparation of the SiO_2_-IO Nanostructures

The SiO_2_-IO nanostructures were prepared by following the previous literature [31]. The silicon wafer substrate was cleaned by washing with EtOH, followed by immersion in piranha solution, a mixture of sulfuric acid (H_2_SO_4_), and hydrogen peroxide (H_2_O_2_) in a 3:1 ratio for 30 min and then in 5% hydrogen fluoride for 15 min. After washing the wafer, it was vertically immersed into the colloidal suspension. The suspension was composed of a 300-nm colloidal polystyrene (PS) aqueous solution and a hydrolyzed TEOS solution. The TEOS solution consisted of TEOS, 0.1 M HCl, and EtOH in a weight ratio of 1:1:1.5. The colloidal suspension was evaporated slowly at 65 °C for 3 days. Then, the stacked colloidal PS on the substrate was removed by calcination in a furnace at 500 °C for 2 h. 

### 2.3. Characterization of the SiO_2_-IO Nanostructures 

The morphology of SiO_2_-IO nanostructures was analyzed by scanning electron microscopy (SEM, Quanta 250 FEG, FEI, Hillsboro, OR, USA) with an acceleration voltage of 10 kV after applying an Au coating. The surface wettability of opal and the IO nanostructure was measured by a contact angle analyzer (Phoenix 300 Plus, SEO Co., Ltd., Suwon, Korea). The reflectance was measured by a spectrometer (FLAME-S, Ocean Optics, Largo, FL, USA). The spectrometer was fixed on an optical table, and the reflection was calibrated by a total reflection mirror, which reflects 100% of the light from 400 to 750 nm.

### 2.4. Surface Functionalization of SiO_2_-IO for Antibody Immobilization 

Surface functionalization was performed through coating with APTMS, conjugation with a cross-linker, and immobilization of antibodies. The first SiO_2_-IO nanostructures were functionalized with APTMS by amine group exposure [32]. SiO_2_-IO nanostructures with exposed hydroxyl groups were immersed in 0.2% ATPMS solution in anhydrous toluene under a nitrogen atmosphere for 12 h. The IO nanostructures were then washed sequentially with toluene, a mixture of toluene and methanol, and methanol. After drying at 80 °C for 30 min to remove the methanol, the second IO nanostructure was conjugated by NHS-PEG_4_-maleimide (19 mM in PBS) for 30 min at room temperature to create cross-links between the protein and the nanostructures. Then, 0.1 mg/mL of Cys-ProG was conjugated to the surface by a maleimide linker for 60 min. Finally, the influenza A (H1N1) virus capture antibody (αHA-1, 1 µg/mL, 200 µL) was applied to the Cys-ProG-conjugated IO surface for 2 h. The sample was washed with PBS buffer between steps.

### 2.5. Detection of the Influenza Virus by the SiO_2_-IO Nanostructures

To detect the influenza A (H1N1) virus using SiO_2_-IO nanostructures, 10 µL of the virus solution was added to the αHA-1 antibody-immobilized Ab-SiO_2_-IO for 2 h. After washing with PBS buffer to remove unreacted viruses, the variation in the reflectance of the SiO_2_-IO nanostructures was measured. No blocking process was applied to the surface before virus detection. Viruses did not directly bind to the Cys-ProG-immobilized SiO_2_-IO nanostructures [21]. Additionally, we confirmed the specificity of the functionalized inverse opal structures. We treated the structure with the H1N1 subtype, the H3N2 subtype, and IFVB, and measured the reflectance change.

To confirm detection of the virus, we prepared αHA-1-functionalized AuNPs. We synthesized AuNPs by the citrate reduction method with slight modifications [33]. Then, 900 µL of the AuNPs (18 nm) were mixed with 100 µL of the GBP-ProG complex (1 mg/mL) overnight at room temperature. The solution was centrifuged twice at 12,000× rpm for 10 min for the washing process. The pellet was dispersed in 0.1× PBS buffer (containing 0.01% Tween20 buffer). In addition, 100 µL of the antibody (0.1 mg/mL) was conjugated with 900 µL of the functionalized AuNPs for 2 h at room temperature. After washing, the antibody-immobilized AuNPs were treated to confirm the virus detection of the IO nanostructures for 2 h. After rinsing with PBS, the SiO_2_-IO nanostructures were analyzed by scanning electron microscopy (SEM, Quanta 250 FEG, FEI, Hillsboro, OR, USA).

## 3. Results and Discussion

### 3.1. Morphologies of the SiO_2_-IO Nanostructures

**Figure 1:**
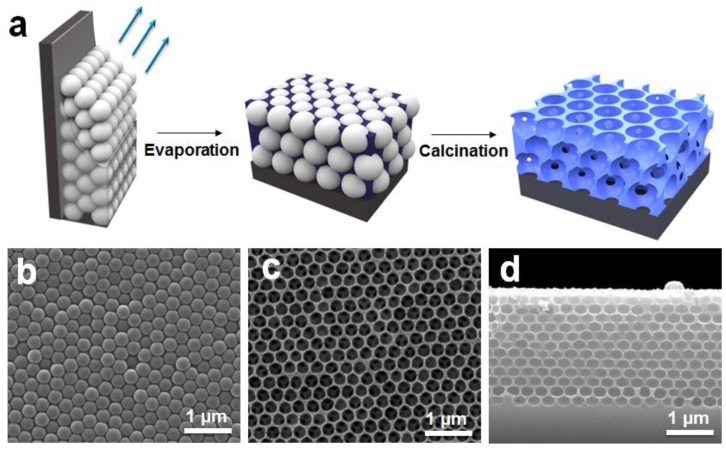
(**a**) Schematic illustration showing fabrication procedure of inverse opal (IO) nanostructure. SEM images of (**b**) the top surface of opal; (**c**) the top surface of the IO nanostructure; and (**d**) the cross-section of the IO nanostructure.

IO nanostructures were generally prepared using an infiltration method in which the matrix materials, such as liquid- [25,34] or gas-phase [35,36,37] precursors, are infiltrated into the interstitial voids of colloidal arrays. After solidification of the matrix, the colloidal particles were removed by heat treatment or chemical etching. However, this method has disadvantages such as the formation of cracks, vacancies, and other defects. To overcome these drawbacks, we used the co-assembly deposition method in which colloidal PS beads and matrix precursors were simultaneously employed to form a film. Figure 1a shows the scheme for the preparation of the SiO_2_-IO nanostructures. First, a colloidal dispersion of PS beads containing TEOS was vertically deposited on the silicon (Si) substrate. When evaporated at 65 °C, the PS beads were periodically arranged to form a closely-packed array by capillary force on the Si substrate, whereas TEOS filled the interstices among the PS beads by forming solid SiO_2_ through a sol-gel process. In general, cracking in opal or IO nanostructures is caused by local capillary forces during drying [38]. However, the TEOS-based sol-gel process can reduce the number of defects by forming a network between the spheres [31,39]. Then, the colloidal PS beads were removed by calcination at 500 °C. The PS beads were replaced by air in the structure, forming the IO nanostructures. 

The morphology of the fabricated opal and SiO_2_-IO nanostructures was characterized by SEM. Figure 1b shows the honeycomb arrangement of colloidal PS beads, which corresponds to (111) planes of a face-centered-cubic (FCC) structure. The silica precursor was solidified by a sol-gel process during the vertical deposition accompanied by evaporation at a constant temperature. During the subsequent calcination step, the PS beads were completely removed, while the SiO_2_ matrix remained undistorted, as seen in Figure 1c. The cavity diameter of the SiO_2_-IO nanostructures was approximately 263.6 ± 12.9 nm, which is smaller than the size of colloidal PS beads, indicating that structural shrinkage of the SiO_2_ matrix occurred during the calcination. The thickness of the SiO2-IO nanostructures on the substrate was 2.46 µm with approximately 12 layers of spherical cavities (Figure 1d). These SEM images indicated that SiO_2_ based IO nanostructures were successfully fabricated by the co-assembly method. 

### 3.2. Optical and Surface Properties of the SiO_2_-IO Nanostructures 

**Figure 2:**
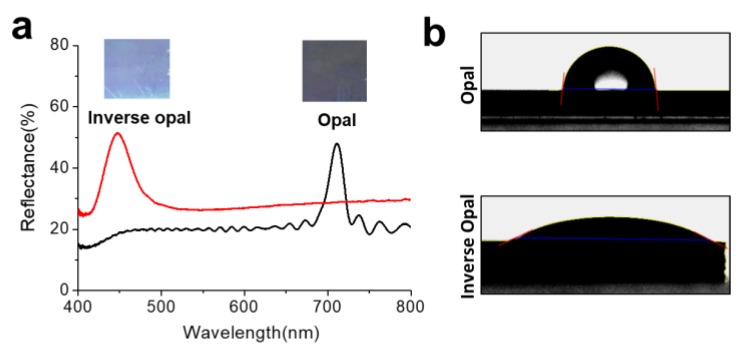
(**a**) Reflectance spectra of the opal and IO nanostructures. Insets are the corresponding optical microscope images; (**b**) Optical images showing contact angles of a water drop on the opal (top panel) and IO (bottom panel) nanostructures.

The optical properties of IOs vary depending on the refractive index and filling factor of the medium. The stop band wavelength, or the reflectance peak position, of the IO nanostructure could be estimated by Bragg’s law for (111) stacked planes of the FCC lattice [40].

(1) $$\lambda =2dn_{eff}=\sqrt{\frac{8}{3}}D\;n_{eff}$$

(2) $$n_{eff}=\sqrt{\phi_{p}n_{p}^{2}+\left(1-\phi_{p}\right)n_{m}^{2}}$$

where *d* is the (111) plane spacing, *D* is the particle diameter, and $n_{eff}$ is the effective refractive index. $\phi_{p}$ is the volume fraction of the FCC colloidal particles ($\phi_{p}$ = 0.74). $n_{p}$ and $n_{m}$ are the refractive indices of the template and the matrix. The opal nanostructures with *D* = 287.21 nm are expected to have a stop band at 727.97 nm from the Equation (1) with refractive indices of polystyrene (1.59) and SiO_2_ (1.45). The IO nanostructures with *D* = 263.6 nm are expected to have the stop band at 488.27 nm with refractive indices of air (1.00) and SiO_2_ (1.45); the cavity diameter was decreased by calcination as we had discussed [41]. In the reflectance spectra experimentally measured, the opal nanostructures showed a reflectance peak at 710 nm and the IO nanostructure showed a peak at 447 nm, as shown in Figure 2a, which are comparable with the stop band positions anticipated from the Bragg’s law. It is also clearly shown that the opal structure is faint gray as the peak is located in near-infrared and the IO structure is shown in sky blue (see the optical images in Figure 2a). Because the reflectance peak position can be varied depending on the refractive index of the surrounding environment, the SiO_2_-IO nanostructures can serve as sensing materials. To confirm the influence of refractive index on the reflectance spectrum, SiO_2_-IO nanostructures were infiltrated with a set of glycerin-water mixtures. Glycerin is a simple polyol compound and viscous liquid, and the refractive index of the mixture depends on the glycerin-to-water ratio. The refractive index increases as the weight percent of glycerin increases [42]; the refractive indices were 1.33, 1.36, 1.38, and 1.41 for 0, 20, 40, and 60 weight percent of glycerin, respectively. Figure S1 shows a correlation between the refractive index of the infiltration liquids and the reflectance peak position. The reflectance peak was redshifted along with the refractive index of the mixture for of the IO with constant volume and cavity size. 

We also measured the contact angle to determine the wettability of the solid surface of the opal and SiO_2_-IO nanostructures. The contact angle is influenced by the surface property of the nanostructures. The contact angle on the opal structure was 82.9° due to hydrophobic colloidal PS beads on the surface of the structure, while that on the SiO_2_-IO nanostructures was 26.33° due to the partially hydrophilic silica matrix (Figure 2b). After APTMS treatment, the contact angle was slightly increased to 37.38° (data not shown) because the hydroxyl groups were eliminated by oxane bonding and the less-polar organic silanes were deposited on the surface [43]. The amine groups on APTMS were used to conjugate biomolecules on the surfaces of cavities.

### 3.3. Surface Functionalization of the SiO_2_-IO Nanostructures

**Figure 3:**
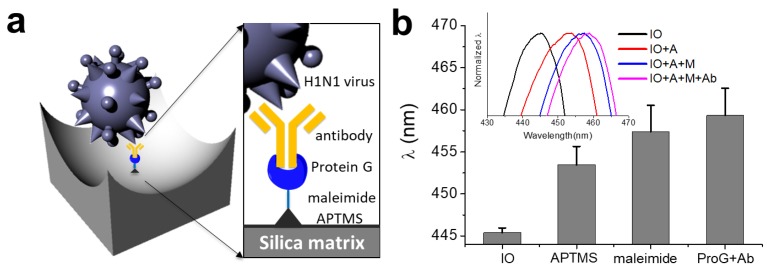
(**a**) Schematic illustration showing the molecular structures formed by the surface functionalization on the IO nanostructure for binding the H1N1 subtype; (**b**) Reflectance peak positions for the pristine, APTMS-treated, NHS-PEG_4_-Maleimide cross linker-treated, and Cys-ProG -antibody immobilized IOs. Inset shows reflectance spectra for all four samples. APTMS: 3-aminopropyl trimethoxysilane.

To detect the influenza A (H1N1) virus, SiO_2_-IO nanostructures were functionalized as illustrated in Figure 3a. First, the silica surface was functionalized by APTMS to exposure amine groups via siloxane bonding. Then, the IO nanostructure was conjugated with NHS-PEG_4_-maleimide, which is a heterobifunctional cross-linker, to link the amine-functionalized surface to another molecule containing a thiol group. Cys-ProG was previously used for the highly efficient immobilization of the immunoglobulin-binding protein (i.e., IgG) on a gold surface [44]. In the same manner, we conjugated Cys-ProG via the maleimide linker for immobilization of the hemagglutinin (HA) antibody on the IO surface. The surface modification method can provide more stable conjugation sites than the physical absorption method. A previous study reported the detection of targets by electrostatic interactions between the negatively charged, porous TiO_2_ surface and the positively charged protein (lysine and/or arginine residues) [27]. In this work, a high concentration of proteins and antibodies (approximately 0.5~2.5 mg/mL) was used in the experiments. To evaluate the surface functionalization of the SiO_2_-IO nanostructures, we measured a series of the reflectance spectra during the modification. As shown in Figure 3b, the reflectance peak was redshifted from 445.41 ± 0.55 nm by approximately 8.05 nm during the APTMS treatment. In addition, the reflectance was further shifted by 3.96 nm and 1.91 nm after treatment with the maleimide linker and Cys-ProG-HA antibody, respectively. The redshifts are results of successful deposition of the molecules on the cavities which slightly increases the refractive index of the medium. 

Antibody immobilization was further confirmed by the HRP activity (Figure S2). Peroxidase consists of a large family of enzymes and catalyzes the oxidation of the substrate with hydrogen peroxide (H_2_O_2_). Peroxidase is widely used in bioanalytical chemistry, for example, to catalyze the conversion of chromogenic substrates, such as 3,3′,5,5′-tetramethylbenzidine (TMB), into colored products [45]. TMB changes to a blue-colored product in the presence of hydrogen peroxide (H_2_O_2_) [46]. The surface functionalization was demonstrated by an HRP-tagged antibody. First, we observed the activity of the functionalized surface. Cys-ProG binds to the antibody through the heavy chains in the region of the Fc fragment. The Cys-ProG-immobilized IO surfaces contain more antibodies than the APTMS-functionalized surfaces. We also compared a SiO_2_ thin film with the surface-functionalized inverse opal. Both substrates have hydrophilic hydroxyl groups; however, the IO structure had higher activity (56.5%) than the SiO_2_ thin film (19.2%) due to its large surface area.

### 3.4. Detection of the Influenza H1N1 Virus by SiO_2_-IO Nanostructures

**Figure 4:**
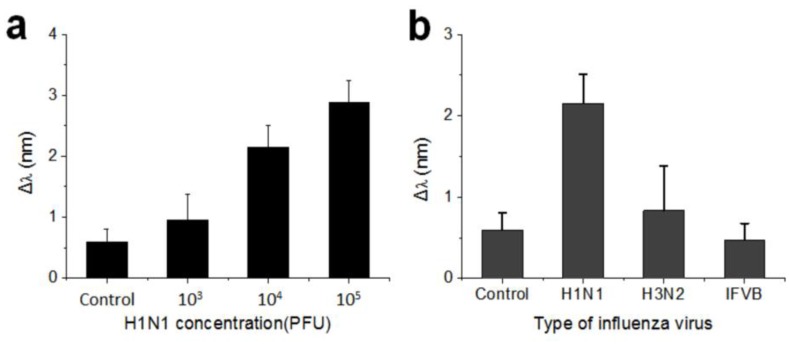
(**a**) The magnitude of reflectance peak shift as a function of H1N1 subtype concentration, where the concentration was varied in the range of 10^3^ to 10^5^ PFU in 10 μL (*n* = 3). Phosphate-buffered saline (PBS) buffer solution is used for the control; (**b**) The magnitude of reflectance peak shift depending on the type of virus, where the concentration was set to 10^4^ PFU (*n* = 3) for influenza A virus subtypes H3N2 and H1N1, as well as the influenza B virus (IFVB).

We investigated the detection of the pandemic influenza type A (H1N1) virus (A/CA/07/2009) by reflectance measurement. The virus is 80~100 nm in diameter and can be classified into 16 hemagglutinin (HA) subtypes and 9 neuraminidase (NA) subtypes [1]. HA is one of the major surface glycoproteins of the H1N1 subtype and is more prevalent than NA on the viral surface [47]. The viruses were detected by monitoring the immune response between the HA and αHA-1 antibodies. We treated 10 µL of the H1N1 subtype with the antibody-immobilized IO nanostructure by concentration. Figure 4a shows that reflectance peak shift as much as 0.96 ± 0.42 nm, 2.15 ± 0.35 nm, and 2.88 ± 0.36 nm that were measured for the concentration range from 10^3^ PFU to 10^5^ PFU on the functionalized IO nanostructure. To evaluate the specificity of the functionalized SiO_2_-IO nanostructures, we compared the H1N1 subtype to the H3N2 subtype and IFVB, each with a concentration of 10^4^ PFU. SiO_2_-IO nanostructures were prepared by αHA-1 antibody immobilization. The control was treated with PBS buffer solution instead of the virus sample. Figure 4b shows the magnitudes of the redshift of the reflectance peak depending on the type of virus. The H3N2 subtype and IFVB showed a shift that is comparable to the control. Only the H1N1 subtype showed a meaningful magnitude of peak shift, confirming that the antibody-immobilized SiO_2_-IO nanostructures have a high specificity to the H1N1 subtype.

**Figure 5:**
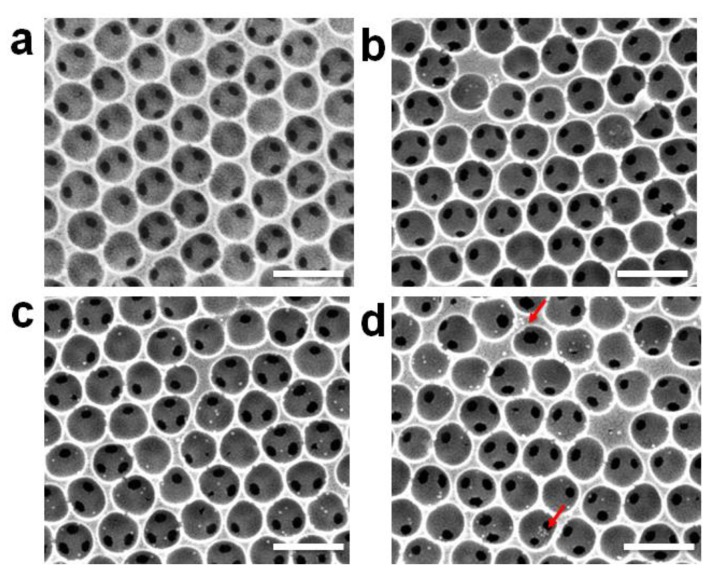
(**a**–**d**) SEM images showing the top surface of the IO nanostructure, where IO is treated with no virus (**a**), or 10^3^ PFU (**b**), 10^4^ PFU (**c**), or 10^5^ PFU of the H1N1 subtype (**d**); Scale bars denote 500 nm.

To prove our concept, we used αHA-1 antibody-immobilized AuNPs to visually confirm the virus. Because the GBP-ProG complex can be employed on the AuNP surface, functional linkers can be used for a scanometric antibody probe [48]. The αHA-1 antibody-immobilized AuNPs were treated with the various virus concentrations captured by the SiO_2_-IO nanostructures. As seen in Figure 5, the SEM images show an increased amount of AuNPs either on the surface or in the pores of the SiO_2_-IO nanostructures (red arrows in Figure 5d), while there were no AuNPs in the control (Figure 5a). This indicates that virus detection using SiO_2_-IO nanostructures was indirectly confirmed by the αHA-1 antibody-immobilized AuNPs.

## 5. Conclusions

In this study, we reported the surface functionalization of SiO_2_-IO nanostructures with antibodies and their use as biosensors for the detection of influenza viruses. We fabricated the SiO_2_-IO nanostructures by the colloidal self-assembly method through vertical deposition and calcination processes. For the detection of influenza viruses, we successfully modified the surface of the SiO_2_-IO nanostructures with antibodies via chemical and biological conjugation. The antibody-immobilized SiO_2_-IO nanostructure captures the target viruses of the H1N1 subtype when immersed in the dispersion of the viruses, which leads to a redshift of reflectance peak. Therefore, the H1N1 subtype can be simply detected by monitoring the redshift of reflectance peak position in the absence of any labeling procedures. We found that the H1N1 subtype was sensitively and selectively detected in the concentration range from 10^3^ to 10^5^ PFU. This result was further confirmed by antibody-immobilized AuNPs. The SiO_2_-IO biosensors have the advantage of large surface area compared with existing optical biosensors due to the three-dimensional nanostructure and enable integration with microfluidics and lab-on-a chip technologies to improve the sensor for point-of-care biosensors. Moreover, various sensing strategies can be employed through the surface functionalization protocol. Therefore, we believe that the SiO_2_-IO nanostructure-based biosensors can be further utilized for the detection of various biohazards.

## Supplementary Materials

The following are available online at http://www.mdpi.com/1424-8220/18/1/307/s1.
