# Supplementary material for: An Antibody-Immobilized Silica Inverse Opal Nanostructure for Label-Free Optical Biosensors
Source: Sensors (Basel). 2018 Jan 20;18(1):307. doi: 10.3390/s18010307 (PMC5796272; doi:10.3390/s18010307)
Supplement: Supplementary file 1 [file sensors-18-00307-s001.pdf]

sensors

Supplementary Information

An Antibody-Immobilized Silica Inverse Opal Nanostructure for Label-Free Optical Biosensors

Wang Sik Lee [1,2], Taejoon Kang [1,2,3], Shin-Hyun Kim [4] and Jinyoung Jeong [1,2,3,*]

1 Hazards Monitoring Bionano Research Center, Korea Research Institute of Bioscience and Biotechnology (KRIBB), 125 Gwahak-ro, Yuseong-gu, Daejeon 34141, Korea; wang3026@kribb.re.kr (W.S.L.); kangtaejoon@kribb.re.kr (T.K.)
2 Department of Nanobiotechnology, KRIBB School of Biotechnology, University of Science and Technology, Daejeon 34113, Korea
3 BioNano Health-Guard Research Center, Global Frontier Project, 125 Gwahak-ro, Yuseong, Daejeon 34141, Korea
4 Department of Chemical and Biomolecular Engineering, Korea Advanced Institute of Science and Technology (KAIST), 291 Daehak-ro, Yuseong-gu, Daejeon 34141, Korea; kim.sh@kaist.ac.kr
* Correspondence: jyjeong@kribb.re.kr; Tel.: +82-42-879-8454; Fax: +82-42-879-8596

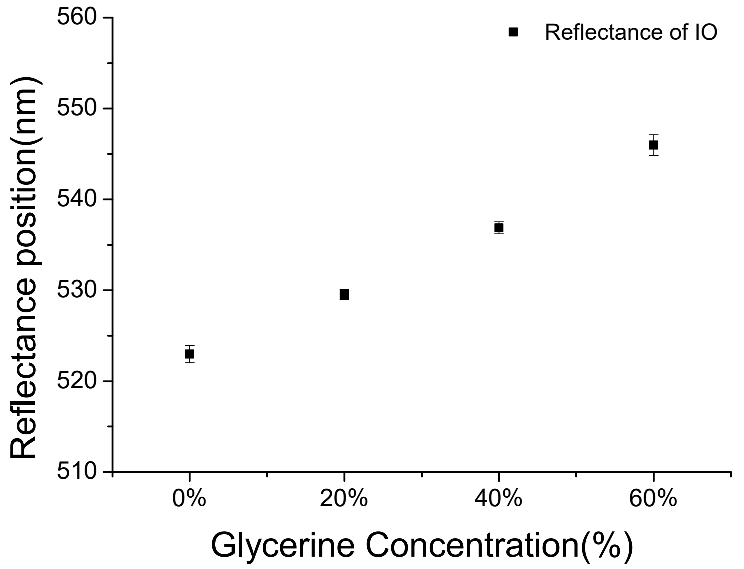

Figure S1. Reflectance peak position of IO nanostructure by glycerin concentration. Refractive index of glycerin is 1.33, 1.36, 1.38, and 1.41 by concentration 0%, 20%, 40% and 60%.

Characterization of Immobilized Antibody on IO Structures

To confirm the immobilization of antibody, we performed the HRP activity test by goat anti-rabbit IgG H&L. Also, we compared SiO_2 thinfilm to IO nanostructure. The HRP tagged antibody was immobilized for 3 h at 4°C on Cys-porG immobilized IO nanostructure. To reduce the non-specific binding, the surface was treated by 1% BSA for 60min. Antibody immobilized IO nanostructure was immersed in the mixture solution of 800 µM of TMB and 50 mM H_2O_2 in 2 mL PBS. After 5 min, 0.1 mL of 2 M of H_2SO_4 solution was added in the mixture solution to stop the reaction. Then, the absorbance at 450 nm was measured by using UV/Visible spectrometer (DU-800, Beckman Co., Brea, CA, USA).

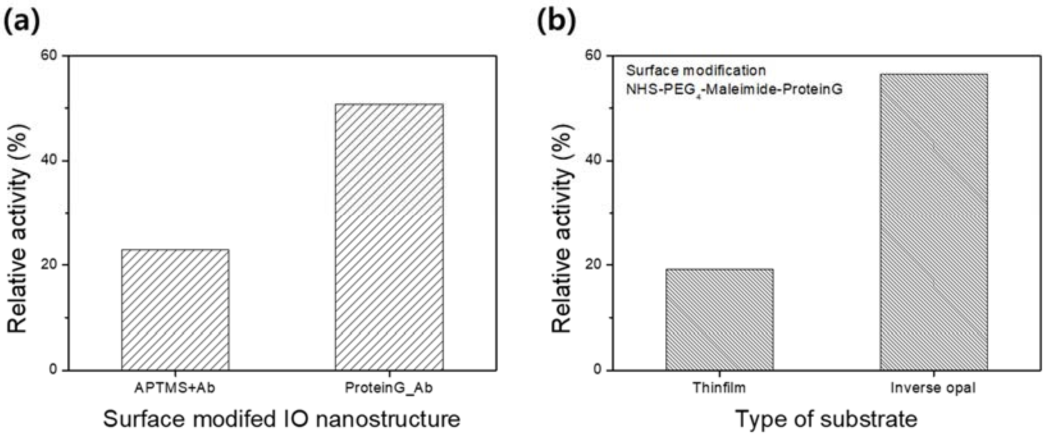

Figure S2. (**a**) HRP activity of IO nanostructure compared APTMS modified IO with proteinG modified IO. (b) Comparison of one-dimensional thin film and three-dimensional IO nanostructure.
